# Isolated Limb Perfusion on Nonmelanoma Skin Cancer for Limb Salvage: A Series of Four Cases

**DOI:** 10.7759/cureus.9998

**Published:** 2020-08-24

**Authors:** Aldo Edyair Jiménez Herevia, Luis Alberto Tavares de la Paz, Diego Hinojosa Ugarte, Jefferson Nieves Condoy

**Affiliations:** 1 Surgery, Bajio Regional High Specialty Hospital, Leon, MEX; 2 Surgical Oncology, Bajio Regional High Specialty Hospital, Leon, MEX

**Keywords:** isolated limb perfusion nonmelanoma, nonmelanoma, skin cancer, squamous cells carcinoma, limb-salvage surgery, isolated limb perfusion

## Abstract

Nonmelanoma skin cancer (NMSC) is more prevalent than all the other cancers combined together. The most common regions affected by skin cancer are the head and neck, but there is a large proportion of the cases located on the limbs. They could be bulky, very extensive and/or located in specialized regions like the hands or near to a joint. Most of those cases should be amputated, with several compromises of the function and a negative impact on the patients' quality of life. Isolated limb perfusion is a proven alternative to limb salvage on soft tissue sarcomas, but there are just a few reports about its application on non-melanoma skin cancer. The aim of the article is to describe the outcomes and prove the benefits and effectiveness to avoid limb amputation when using isolated limb perfusion on locally advanced non-melanoma skin cancer. We present clinical, retrospective study as a case series report. The study includes four patients with locally advanced non-melanoma skin cancer in the limb - three cases with squamous cell carcinoma (SCC), and one patient with Merkel cell carcinoma (MCC) who underwent to tumor necrosis factor-alpha (TNFα) and melphalan based isolated perfusion of the limb (TM-ILP). Toxicity, clinical response, and limb salvage were described. Patients were treated in the oncological surgical department in a referral hospital in Mexico. The limb salvage rate was achieved in 75%. All patients had a favorable response to TM-ILP. Complete response of the tumor was noted on two (50%) of the patients (one with histopathological confirmation), the other 50% had a partial response to the TM-ILP. No serious toxicity related to TM-ILP was observed. One patient (25%) developed regional lymph involvement six months after perfusion. Lymphadenectomy was performed, and the patient subsequently has a six-year disease-free survival. In conclusion, TM-ILP could be an effective, reliable, and safe therapy on limb salvage in selected patients with non-melanoma skin cancer who are not adequate surgical candidates.

## Introduction

According to 2018 GLOBOCAN (global cancer statistics), non-melanoma skin cancer (NMSC) is located in the top ten malignancies worldwide, in the population of Caucasians is more commonly diagnosed than all other malignancies combined. NMSCs represent a diverse group of skin tumors, including cutaneous squamous cell carcinoma (cSCC), basal cell carcinoma (BCC), extramammary Paget's disease (EMPD), Merkel cell carcinoma (MCC), and skin adnexal carcinomas. Of these, BCC and cSCC account for the majority of NMSCs (75-80% and 20-25% of all NMSC cases, respectively). The most common regions affected by skin cancer are the head and neck, but there is a large proportion of the cases located on the limbs [1]. Recommended treatment for NMSC remains to be a surgical excision following a positive biopsy [2]. The most used techniques are tangential shave removal, curettage, electrodesiccation, Mohs micrographic surgery (MMS), and standard surgical excision [3]. The tumor could be very extensive and/or located in specialized regions like the hands or close to a joint or have histopathological and clinical features associated with bad outcomes; most of those cases should be amputated, with several compromises of the function and a negative impact on the patients' quality of life. Isolated limb perfusion (ILP) is a proven alternative therapy for limb preservation, especially on patients with soft tissue sarcomas (STS) [4]. There is a shred of large evidence on its favorable results on soft tissue sarcomas of the limbs, and it is considered a feasible and safe alternative to surgery on selected patients [5-7]. There are just a few reports on NMSC cases [8-10]. ILP could be done with one or two chemotherapy agents - melphalan and recombinant human tumor necrosis factor-alpha (TNFα). TNFα is a cytokine, which increases vascular permeability; also, it increases the extravasation of cytotoxic agents and selective lysis of the endothelial by apoptosis and inflammation of the tumor vessels, and when combined with melphalan it could have favorable effects on bulky or well-vascularized tumors [4]. The main outcome is to avoid amputation, improving patient quality of life by the preservation of the limb in patients with unresectable NMSC, even when it's established that the ILP doesn't represent an improvement in survival. The objective of this article is to report our experience with melphalan based isolated limb perfusion (TM-ILP) in NMSC based on case series. 

## Case presentation

Patients and methods

Data were retrospectively collected on all patients treated with ILP between 2010-2020 at the Bajio Regional High Specialty Hospital in Leon, Mexico. The inclusion criteria were patients with NMSC. We eliminated all the patients with melanoma and sarcoma. Only four patients in our cohort had met the inclusion criteria - three patients with cSCC, and one with MCC. Patients prospectively gave informed consent for data collection and usage for future clinical research. The ILP procedure was identically performed in each case by the same surgical team. An identical ILP circuit described by other authors was used.

Description of ILP technique

In this article, we are not focusing on describing the technique in detail, but give an overview of critical points of the technique. With a previous heparinization, the surgeon identifies and cannulates the main vessels of the limb. Then, the vessels should be clamped or occluded in the proximal site. With the use of an extracorporeal circulation pump, the chemotherapy is infused into the limb [11]. Melphalan is titrated with 50 mg/kg for superior extremity and 100 mg/kg for lower extremity, and TNFα is titrated with 1 mg/kg and 2 mg/kg, respectively. The temperature should be between 38° C and 40° C. Tissue temperatures are measured with two thermometers, which are placed in subcutaneous cell tissue and muscle. Systemic and regional toxicity should be assessed during perfusion with a gamma probe and radiomarked albumin on the systemic circulation [12].

Postoperative care and toxicity

Postoperatively, patients were monitored in the intensive care unit for 24 hours and subsequently transferred to the surgical ward. Toxicity to the ILP was measured and recorded by daily physical examination while hospitalized and for three months postoperatively using the scale proposed by Wieberdink et al., which was used in several other studies (see Appendix) [13]. As a prophylactic measure, all of the patients were put on low-molecular-weight heparin on the first day after the ILP procedure and stayed on treatment until hospital discharge.

Case 1

**Figure 1:**
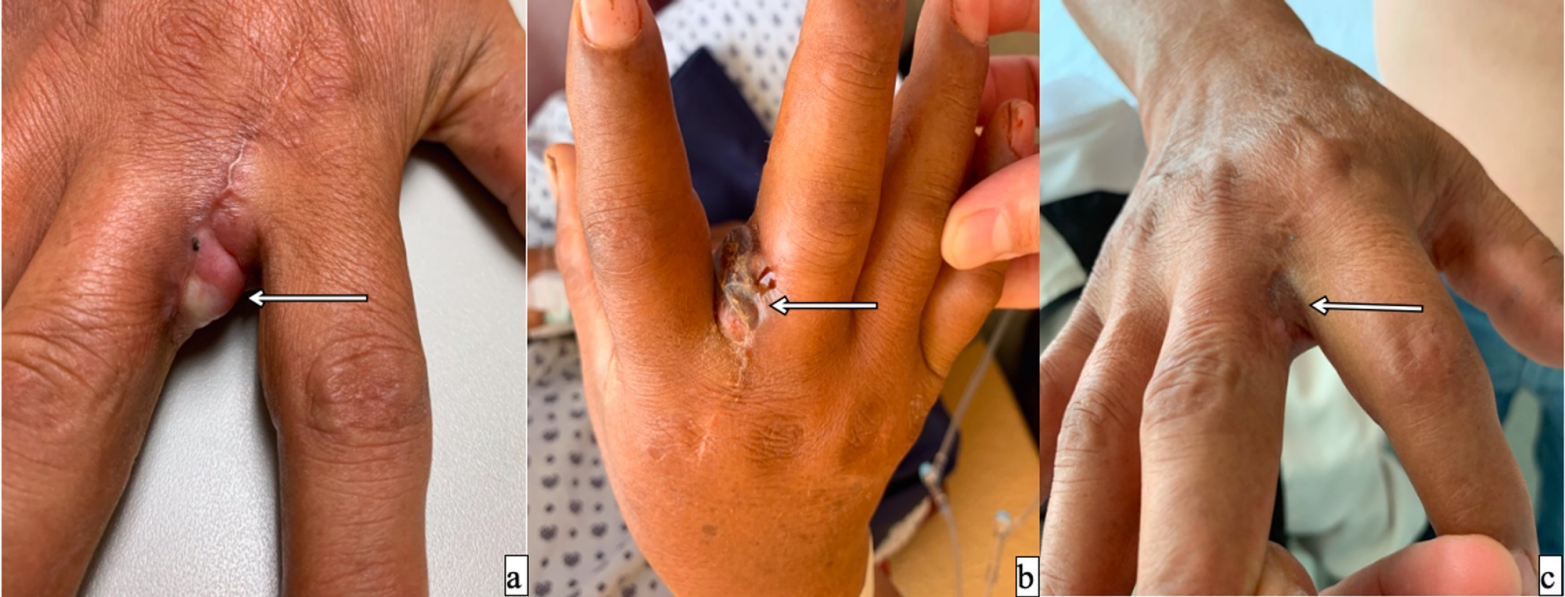
A 63-year-old male with a right hand Merkel cell cancer between the second and third fingers a) previous to TM-IPL;  b) ongoing response after two weeks; c) complete response after six months. TM-IPL - melphalan based isolated limb perfusion

A 63-year-old male with a right-hand tumor between the second and third fingers was treated by a non-oncologist surgeon. Surgical excision was performed; the histopathological report was consistent with MCC and R1 resection. Axillary lymphadenectomy with ILP was performed (temp 38.5-39º C, TNFα 15 min, melphalan 35 min, leak inferior to 5%), no adverse effects were observed (Wieberdink I), and the patient stayed in the intensive care unit (ICU) for 24 hours. In the post-surgery histopathological report, 7/7 axillary lymph nodes were reported as follicular lymphoid hyperplasia. After 13 months, no local recurrence was observed, and the limb was salvaged with preserved total functionality (Figure 1).

Case 2 

**Figure 2:**
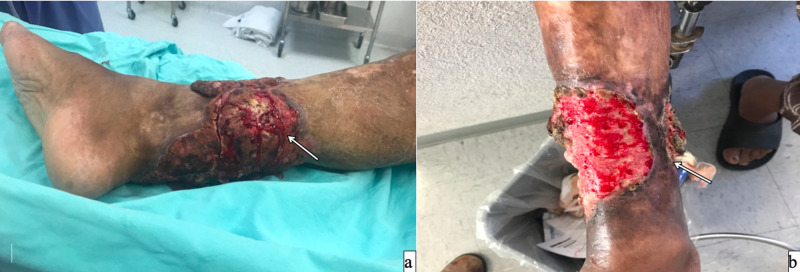
A 58-year-old male's right leg with a squamous cell cancer with recurrent infection a) previous to TP-ILP; b) ongoing response after two weeks. The patient required amputation due to the persistence of the infection. TM-IPL - melphalan based isolated limb perfusion

A 58-year-old male with a previous history of type 2 diabetes mellitus, with a burn injury of 40 years ago on his right leg, developed a Marjolin's ulcer and finally, a high-grade squamous cell cancer. He underwent at least three surgical resections and a skin graft repairment. The tumor had local recurrence, and it took more than 70% of the leg's circumference. The tumor was friable, exophytic, and necrotic, with a full-thickness (including bone) compromise of the limb (Figure 2). The patient had recurrent infections of the tumor, and a double scheme with antibiotics was administered. The perfusion was done (temp 39-40º C, infusion rate 500 mL/min, maximum leak of 1.4%, TNFα 15 min, melphalan 30 min). The patient had minimal adverse effects (Wieberdink II), stayed for 24 hours in the ICU and was discharged five days later. Fifty-four days after, the patient continues with persistent infection and poor response to antibiotics, finally, supracondylar amputation was decided.

Case 3

**Figure 3:**
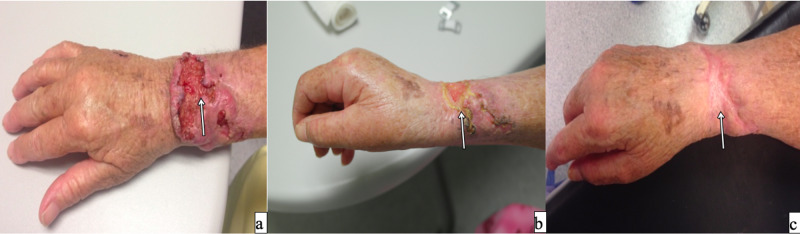
A 71-year-old female with SCC on her right wrist Because of the important location and impact of the function of the hand, it is considered unresectable. TM-ILP was done. a) tumor previous to TM-ILP; b) ongoing response after two weeks; c) complete response of the tumor after four months of TM-ILP. SCC - squamous cell carcinoma; TM-IPL - melphalan based isolated limb perfusion

A 71-year-old female developed a right wrist low-grade squamous cell cancer with infiltration to the deep fascia of the right-hand dorsum. Isolated perfusion was done (infusion rate 200-350 mL/h, leak less than 1%, TNFα 20 min, melphalan 45 min) with no adverse effects (Wieberdink II). The patient was 24 hours in the ICU. A posterior biopsy was taken, with dense fibrotic and necrotic tissue, no residual tumor was reported. The patient had a regional recurrence on the axillary lymph nodes six months later. A lymphadenectomy followed by radiotherapy was decided, with metastatic disease on 2/23 of the lymph nodes. Six years after the procedure, the patient continues disease-free status (Figure 3).

Case 4

**Figure 4:**
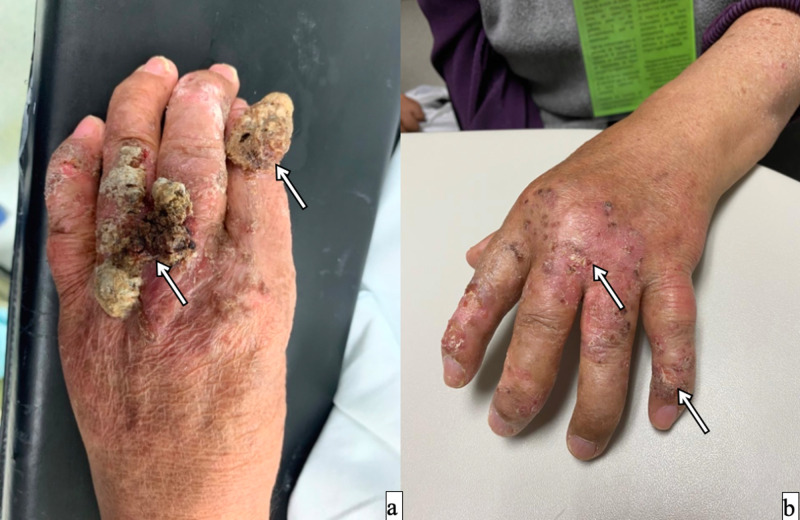
A 76-year-old female with a SCC on her left hand A resection of metacarpal region, second, third and fourth finger could be done with several compromise function of the hand. A TM-ILP was done. a) tumor previous to perfusion; b) complete response after six months. SCC - squamous cell carcinoma; TM-IPL - melphalan based isolated limb perfusion

A 76-year-old female with a history of type 2 diabetes mellitus, noncontrolled systemic hypertension, developed a tumor in the dorsum of second, fourth, and fifth fingers on the left hand. Perfusion was done (leak rate lesser than 2.4%, TNFα 10 min, melphalan 20 min). The patient stayed in the ICU for 24 hours with no adverse effects (Wieberdink I) and was discharged three days later. The patient had a clinical response about 60% of the tumor size, with an adequate function of the hand (Figure 4). Four months later, the patient had a new onset of tumoral activity on the preauricular and nasal dorsum region. 

Table 1 summarizes the four cases presented in this report. 

**Table 1:** Demographic, technical and clinical response characteristics of patients submitted to ILP LT - local toxicity (according to Wieberdink classification); Mel - melphalan; MCC - Merkel cell cancer; cSCC - cutaneous squamous-cell cancer; ILP - isolated limb perfusion; TNFα - tumor necrosis factor-alpha

#	Gender	Age (years)		Location	Size (mm)	Histology type	Artery used	Perfusion duration	LT	Clinical response
1	M		63	Right hand	15 x 20	MCC	Axillary	TNFα 15 min; Mel: 35 min	I	Complete
2	M		58	Right leg	150 x 100	cSCC, G3	Femoral	TNFα: 15 min; Mel: 30 min	II	Partial
3	F		71	Right hand	20 x 8	cSCC, G1	Brachial	TNFα: 20 min; Mel: 45 min	II	Complete
4	F		76	Left hand	30 x 10	cSCC, G2	Axillary	TNFα: 10 min; Mel: 20 min	I	Partial

## Discussion

The main purpose of this article was to present our experience in particular cases of cSCC. All four patients were treated with ILP, all the patients had a favorable response to the perfusion, with a complete response of 50%, and a partial response of 50%, and low systemic and local toxicity. In one patient, the initial response was observed, but an amputation had to be done 54 days after the procedure due to a persistent infection of the tumor, so the whole potential response to IPL couldn't be evaluated. One patient was biopsied after the ILP because of the apparent no change in the tumor size, revealing a complete remission of the neoplastic activity (reported as dense fibrotic and necrotic tissue with no neoplastic cells). One patient had regional tumoral activity, and an axillary lymphadenectomy was made concurrent with ILP. Another patient developed regional activity six months later, and an axillary lymphadenectomy was done, with a six years disease-free status. We consider that a concomitant lymphadenectomy with ILP could be considered a good strategy to avoid a second surgical procedure and to take advantage of the surgical site when locating the main vessel in ILP in selected patients. The rest of the patients had a favorable clinical outcome - two of them with a disease-free outcome, and the other one with a partial reduction of about 60% of the tumor size. 

In our series, no patients presented serious adverse effects related to ILP (Wieberdink I 50%, Wieberdink II 50%). We assumed that this scenario is attributable because of the low leak rate while the perfusion was performed (see Table 1). All patients were in the ICU on the first day to monitoring cardiovascular, renal, and electrolytic impairment or any other local or systemic adverse effects, and all were discharged from the ICU 24 hours later.

The ILP is a proven salvage alternative to surgical resection in soft tissue sarcomas (STS). There are low evidence and reports of TM-ILP in patients with NMSC [9-11]. A recent article of TM-ILP on NMSC with a case series of 30 patients reported an overall response rate of 81%, with a complete response rate of 59%, and a limb salvage rate of 80%, with almost 50% of disease-free status patients, another article reported 80% rate in limb salvage when using TM-ILP [8]. In this report, we found a 75% of limb salvage, with a regional recurrence of one patient (25%) who underwent surgery (axillary lymphadenectomy). 

## Conclusions

Even when there are several reports about ILP as an effective therapy to limb salvage or palliative treatment for STS, there are just a few reports of ILP on NMSC. Although this case series has only four cases, to our knowledge this is the first article in the use of TM-ILP on NMSC reported in Latin America. Also, we expect that this case series is helpful in the future for making decisions and contributes to the literature. It is very important to consider the tremendous physical and psychological impact on a patient when an amputation is performed. It carries very serious limitations on the lifestyle and comfort of the patient, sometimes with a poor prognosis after surgical treatment. Most of the surgeries on patients with a limb tumor or a special area like a joint or hand would get mutilated or involve a severe compromise of their functional status, often with poor or no impact on their survival. IPL could be used as a reliable and safe alternative for limb salvage on selected patients who are not candidates to a surgical procedure.

## Appendices

**Table 2:** Wieberdink's local toxicity classification

Type	Description
1	No subjective or objective evidence of reaction.
2	Slight erythema and/or edema.
3	Considerable erythema and/or edema with some blistering; slightly disturbed motility permissible.
4	Extensive epidermolysis and/or obvious damage to the deep tissues, causing definite functional disturbances; threatening or manifest compartmental syndromes.
5	A reaction, which may necessitate amputation.
